# Pressure shock fronts formed by ultra-fast shear cracks in viscoelastic materials

**DOI:** 10.1038/s41467-018-07139-4

**Published:** 2018-11-12

**Authors:** M. Gori, V. Rubino, A. J. Rosakis, N. Lapusta

**Affiliations:** 1 Graduate Aerospace Laboratory (GALCIT), California Institute of Technology, Pasadena, CA 91125 California (CA) USA; 2 Mechanical and Civil Engineering, California Institute of Technology, Pasadena, 91125 CA USA; 3 Seismological Laboratory, California Institute of Technology, Pasadena, CA 91125 California (CA) USA

## Abstract

Spontaneously propagating cracks in solids emit both pressure and shear waves. When a shear crack propagates faster than the shear wave speed of the material, the coalescence of the shear wavelets emitted by the near-crack-tip region forms a shock front that significantly concentrates particle motion. Such a shock front should not be possible for pressure waves, because cracks should not be able to exceed the pressure wave speed in isotropic linear-elastic solids. In this study, we present full-field experimental measurements of dynamic shear cracks in viscoelastic polymers that result in the formation of a pressure shock front, in addition to the shear one. The apparent violation of classic theories is explained by the strain-rate-dependent material behavior of polymers, where the crack speed remains below the highest pressure wave speed prevailing locally around the crack tip. These findings have important implications for the physics and dynamics of shear cracks such as earthquakes.

## Abstract (web summary)

Propagating shear cracks in solids emit both shear and pressure waves, but it is usually thought that only shear waves coalesce to form shock fronts when the crack exceeds the shear wave speed. Here, the authors show that local material stiffening can further increase rupture speed and produce pressure shock fronts that hint at supersonic propagation.

## Introduction

Shock fronts are sharp discontinuities that arise whenever a perturbing feature, such as a crack traveling through a medium, exceeds the characteristic speed of the waves by which the energy is transferred in the medium. In such a situation, the waves coalesce into a sharp shock front, as observed in atmospheric supersonic flight, hypersonic re-entry from space, meteoroid transit through the atmosphere, and motion of planets with respect to the solar wind^1–5^.

In fracture mechanics and geophysics, shear shock fronts have been observed to arise by the coalescence of shear waves emitted by tips of spontaneously propagating shear ruptures exceeding the shear wave speed of the surrounding material^6–9^. These ruptures are commonly referred to as intersonic or supershear. The speed of the spontaneously propagating cracks is a fundamental problem that has captivated the interest of the scientific community for several decades due to its implications across multiple scientific and engineering disciplines^6,9–18^. In particular, the study of shear cracks propagating along frictional interfaces and the associated shock fronts is relevant to earthquake dynamics^19–22^. The formation of the shock fronts is an important problem in its own right, due to implications of this phenomenon for strong ground motion much farther from earthquake-producing faults than currently accounted for in seismic hazard^6–9,19–21^.

Spontaneously propagating cracks are driven by elastodynamic waves, where the energy released by the crack motion is transferred through the medium to the crack tip region with the (higher) pressure wave speed and (lower) shear wave speed. It is intuitively evident that a crack cannot exceed the fastest way to transfer energy: the pressure wave speed^6,10,11,13,14^. Hence, the formation of a shock front may appear impossible for the pressure waves.

In this study, we provide the first experimental evidence of spontaneously propagating shear ruptures forming a pressure shock front and explain the formation by the strain-rate-dependent—and hence spatially variable—stiffening of the material in the vicinity of the rupture tip. The presence of the pressure shock fronts enables us to refer to our cracks as supersonic.

## Results

### Formation and observation of pressure shock fronts

**Fig. 1:**
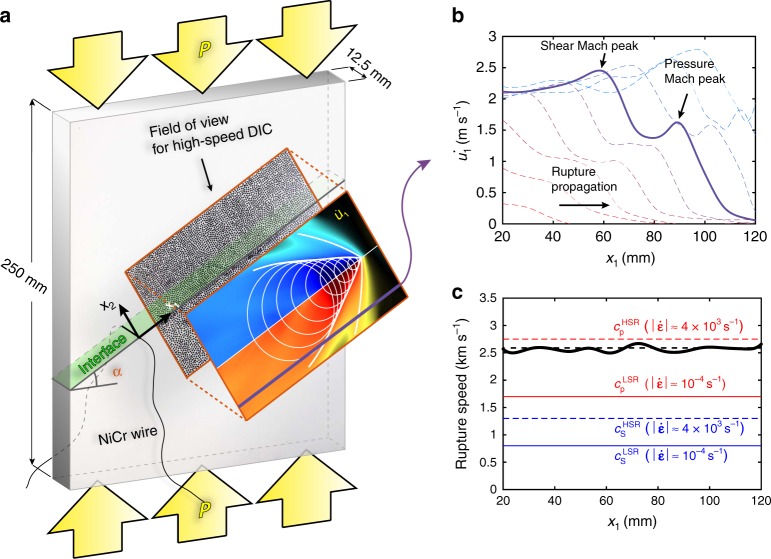
Laboratory set-up and the captured ultra-fast shear ruptures. **a** Dynamic rupture is produced on a sample interface (green-shaded area) loaded in compression and shear by a compressive vertical load (yellow arrows). The rupture is triggered by the sudden disintegration of a Ni-Cr wire filament and subsequently propagates spontaneously over the interface. Its dynamics is captured using a speckle pattern applied over a portion of the specimen’s surface, ultra-high-speed photography, and DIC algorithms. The inset exhibits the distribution of interface-parallel particle velocity, $\dot u_1$, 58 μs after nucleation. The white lines highlight the peaks associated with the pressure and shear shock fronts, and the white circles illustrate how the shear shock front is generated by the coalescence of shear wavelets. **b** The profile of the particle velocity, $\dot u_1,$ along the line (violet) at a distance *x*_2_ = − 27.5 mm from the interface, plotted at time intervals of 5 μs, exhibits two peaks associated with the pressure and shear Mach fronts. **c** The rupture speed vs. position along the interface, *x*_1_, is computed by tracking the rupture tip in the temporal sequence of velocity maps. The comparison with the pressure wave speed $c_{\mathrm{p}}^{{\mathrm{LSR}}}$ for the low strain rates (Fig. 3) confirms the supersonic nature of the rupture, $V_{\mathrm{r}} > c_{\mathrm{p}}^{{\mathrm{LSR}}}$ (see text). At the crack tip, where considerably higher strain rates develop (Fig. 3, lower inset), the rupture is locally intersonic, $c_{\mathrm{s}}^{{\mathrm{HSR}}} < V_{\mathrm{r}} < c_{\mathrm{p}}^{{\mathrm{HSR}}}$

**Fig. 2:**
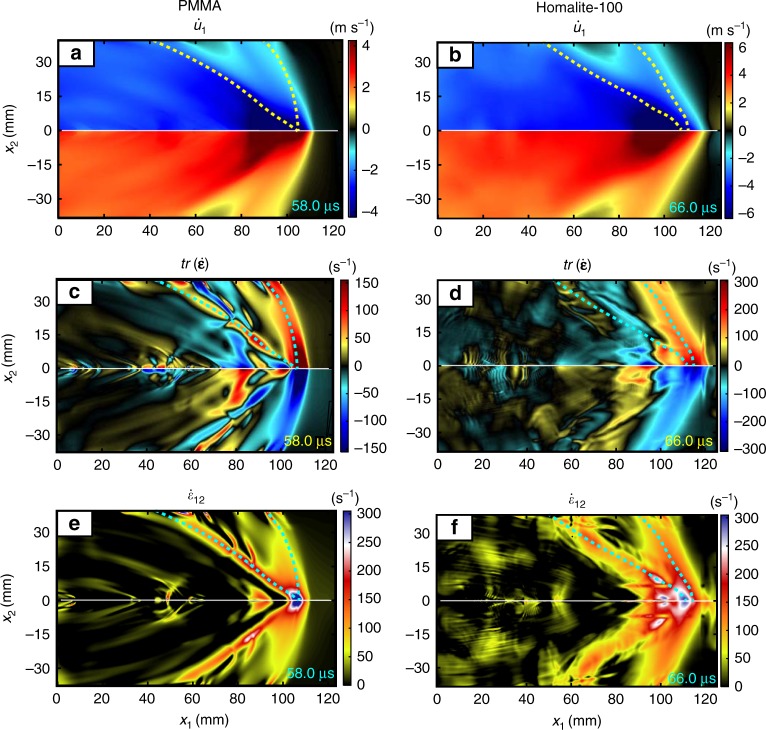
Full-field particle velocities and strain measures for supersonic ruptures. Ruptures in both PMMA (left) and Homalite-100 (right) exhibit two pairs of shock fronts, the pressure and the shear one (colored dashed lines). The PMMA snapshots correspond to 58 μs after the triggering and the Homalite-100 ones to 66 μs. **a**, **b** Interface-parallel particle velocity, $\dot u_1$. **c**, **d** Volumetric strain rate, ${\mathrm{tr}}\left( {{\dot{ \varepsilon }}} \right)$ (see Supplementary Movie 1 on the temporal evolution of the volumetric strain rate in PMMA). **e**, **f** Shear strain rate, $\dot \varepsilon _{12}$. The volumetric strain-rate field (**c** and **d**) clearly shows the presence of the pressure shock front, whereas the shear strain-rate field (**e** and **f**) highlights the shear shock front. The pressure and shear shock fronts, highlighted by dashed lines, are traced as the loci of maxima of the volumetric strain rate (**c**, **d**) and shear strain rate (**e**, **f**), respectively

The presented dynamic shear ruptures are produced in an experimental set-up developed to mimic earthquakes in the laboratory^6,8,9,22^ (Fig. 1a; see Methods section). The set-up features a quadrilateral specimen made of a polymeric material—either Poly(methyl methacrylate) (PMMA) or Homalite-100—with an interface inclined at an angle *α* (Fig. 1a). The uniform external load *P* vertically applied to the specimen (yellow arrows) results in a normal and a shear static pre-stress acting along the interface. The tests exhibited in Figs. 1 and 2 have been conducted under the following experimental conditions: *P* = 21 MPa and *α* = 30° for PMMA and *P* = 25 MPa and *α* = 29° for Homalite-100. To check the repeatability of the experimental outcomes, several tests have been done under nominally the same experimental conditions. The ruptures are triggered by the local brief pressure release due to the rapid sublimation of a Ni-Cr wire filament placed across the specimen’s interface turning into plasma. The rupture initiation is imposed, but the subsequent crack propagation is spontaneous. This laboratory earthquake set-up has been successfully employed in the past to study several key rupture phenomena including supershear transition to intersonic speeds^9^, rupture directionality and limiting speeds due to bimaterial effects^23^, off-fault damage generation^14^, pulse-like to crack-like transitions^24^, opening of thrust faults^25^, and friction evolution^26^.

It is quite challenging to capture the highly dynamic evolution of these shear ruptures in the laboratory, as they take mere tens of microseconds to span the experimental samples. The full-field visualization of supersonic cracks employed here is enabled by our recently developed dynamic imaging technique^26^ based on a combination of high-speed photography and digital image correlation (DIC; see Methods section). A sequence of 128 high-speed digital images—with temporal sampling of up to two million frames per second—is converted into a temporal series of displacement fields by the DIC method, with algorithms tailored to treat displacement discontinuities along an interface^26^. The particle velocity and strain fields are computed by temporal and spatial differentiation of the displacement fields, respectively. The strain-rate maps are obtained by time differentiation of the strain fields. In a set of repeated experiments, an array of four strain-gage stations have been placed along the interface to capture the rupture’s arrival time and to confirm the supersonic nature of the rupture (Supplementary Fig. 1).

The full-field images of the particle velocity, strain, and strain-rate fields during dynamic ruptures in our experiments exhibit two pairs of sharp fronts diverging from the rupture tip, associated with the formation of the pressure and shear shock fronts (Fig. 1a, inset; Fig. 2 and Supplementary Fig. 2; and Supplementary Movie 1). The shear shock fronts, occurring when the rupture exceeds the shear wave speed, have been observed using photoelasticity^6,8,9,14,22,25^, a technique sensitive to shear deformations. Our newly developed high-speed DIC technique reveals the additional formation of a pressure shock front. The pressure shock fronts are most visible in the distribution of the volumetric strain rate, ${\mathrm{tr}}\left( {{\dot{\boldsymbol \varepsilon }}} \right)$ (Fig. 2c, d), whereas the shear shock fronts are most noticeable in the distribution of the shear strain rate, $\dot \varepsilon _{12}$ (Fig. 2e, f), consistent with the properties of the corresponding waves.

### Confirming the shock nature of the pressure features

How can we confirm that these features are indeed pressure shock fronts and not some other expression of a pressure wave field that would be present around any crack tip^8^? As the shock front is the envelope of coalescing waves, the defining feature of a shock front is the kinematic relationship that holds among the inclination angle *β* of the shock front, the wave speed (either *c*_s_ or *c*_p_ depending on the front), and rupture speed *V*_r_^1,5,7–9^:

1 $$\beta _{{\mathrm{s}},{\mathrm{p}}} = \arcsin ( {c_{{\mathrm{s}},{\mathrm{p}}}/V_{\mathrm{r}}} )$$

The inclination angle of the shock front is the angle that the front forms with the path of the propagating feature, in our case the specimen's interface.

To verify this relation for the pressure shock fronts, we need to find the rupture speed, inclination angle, and wave speed of the material. We compute the rupture speed *V*_r_ from the temporal series of velocity maps by tracking the rupture tip location along the interface at each frame (Fig. 1c and Methods section). This leads to the steady rupture speed of *V*_r _= 2.57 km s^−1^ within the window of observation for the experiment with PMMA shown in Figs. 1, 2 (left column), 3 (top inset), Supplementary Figure 2, and Supplementary Movie 1. The inclination angle varies along the pressure shock front (Fig. 2); for the steady rupture speed, the kinematic relation (Eq. 1) would imply that the wave speeds are decreasing in the interface-normal direction. Such an observation is consistent with the viscoelastic response to spatially variable strain rates, with the higher strain rates closer to the crack tip leading to more viscoelastic stiffening and hence higher wave speeds. Indeed, several experimental studies in polymers, including PMMA^18,27–31^ and Homalite-100^7,8,27^, have accounted for their viscoelastic nature by considering the specimens as still uniformly linear elastic but with uniformly altered (stiffer) values of elastic constants during their dynamic response. Some of those studies^29–31^ considered two sets of uniform material properties: unstiffened (low-frequency) ones ahead of the rupture arrival and stiffened (high-frequency) ones for the spatial locations along the interface behind the rupture tip. Several studies^6–8,18,27,28,31^ observed crack tip speeds similar to the ones reported in this work but did not recognize their significance, comparing the crack tip speeds to the uniformly higher dynamic wave speeds and concluding that the cracks are intersonic, a well-known phenomenon^7,8,18,28,31^, where pressure shock fronts cannot exist. Our findings emphasize the qualitative importance of the viscoelastic effects in creating the spatially heterogeneous stiffening—due to spatially inhomogeneous strain rates—that has not yet been considered, which is key to the formation of the pressure shock fronts.

## Discussion

**Fig. 3:**
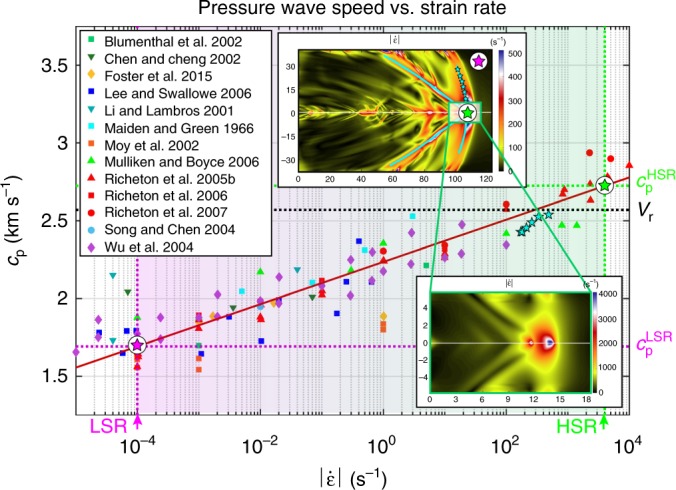
Strain-rate dependence of the pressure wave speed in PMMA. The values of the pressure wave speeds *c*_p_ computed based on the elastic moduli versus strain-rate data acquired from the literature as reported in the legend^32,34–37,48–55^, where the diamond symbols indicate tensile tests, as opposed to the other symbols that indicate compressive tests. The red solid line is a logarithmic fit of this data (see Methods section). The values of *c*_p_ for several locations along the pressure shock front (cyan stars), predicted from the relation (Eq. 1) between the inclination angles of the pressure shock front and rupture speed, are plotted vs. the strain rate determined at those locations. The two sets of pressure wave speeds (from the literature and from our experiments) show an excellent agreement, demonstrating that the inclination angles of the pressure shock fronts are consistent with the viscoelastic stiffening induced. The rupture speed *V*_r_ = 2.57 km s^−1^ (dotted black line) is higher than all of those shock-wave determined pressure wave speeds, indicating that the rupture is supersonic with respect to them. The high-strain-rate (HSR) pressure wave speed, corresponding to the strain rate of about 4 × 10^3^ s^−1^ at the crack tip (the blue region in the bottom inset), is determined as the wave speed on the logarithmic fit (red line) that corresponds to that strain rate (green star). The strain-rate level at the crack tip is obtained from a similar test performed on a sample at an angle *α* = 29° (rather than 30°) under the same loading conditions, by focusing on a smaller field of view, which enables a higher strain-rate resolution (lower inset). The low-strain-rate (LSR) pressure wave speed (see Discussion section) corresponds to the purple star. The green and purple vertical dashed lines refer to the equivalent strain-rate levels for these near-field and far-field measurements, and the corresponding predicted values of the pressure wave speeds are indicated by the horizontal green and purple dashed lines

We find that the non-uniform stiffening due to viscoelastic effects, and hence spatially variable wave speeds, can indeed explain our experimental observations, including the inclination angles of the pressure shock front observed in our experiments. We use the published data on how strain rates affect the Young’s and shear moduli^32–37^ (Fig. 3), focusing on the PMMA due to the abundance of available data. We employ the approximation of quasi-elastic solid^38,39^ (see Methods section), in which the functional form for the material properties is that of a linear-elastic solid but each effective material constant is assumed to depend on the local, instantaneous level of the strain rate. As a consequence of this approximation, the effective wave speeds of the polymers investigated here are functions of the strain rate^32–37^ (Fig. 3). For the areas not yet reached by the crack or waves, the strain rate is near zero; we select the low-strain-rate (LSR) value of $\left| {{\dot{\boldsymbol \varepsilon }}} \right| = 10^{ - 4}{\kern 2pt} {\mathrm{s}}^{ - 1}$ to represent that regime, and the corresponding pressure and shear wave speeds are $c_{\mathrm{p}}^{{\mathrm{LSR}}} = 1.79{\kern 2pt} {\mathrm{km}}{\kern 1pt} {\mathrm{s}}^{ - 1}$ and $c_{\mathrm{s}}^{{\mathrm{LSR}}} = 0.86{\kern 2pt} {\mathrm{km}}{\kern 1pt} {\mathrm{s}}^{ - 1}$, respectively (Fig. 3 and Fig. 1c). As $V_{\mathrm{r}} \, > \, c_{\mathrm{p}}^{{\mathrm{LSR}}}$ (Fig. 1c), the rupture propagates supersonically with respect to the effective pressure wave speed of the far field, which experiences the LSR conditions. At the crack tip, much higher strain rates—of the order of $\left| {{\dot{\mathrm \varepsilon }}} \right| = 4 \times 10^3{\kern 1pt} {\mathrm{s}}^{ - 1}$—develop (Fig. 3, bottom inset), constituting the high-strain-rate (HSR) regime. The corresponding effective pressure and shear wave speeds are $c_{\mathrm{p}}^{{\mathrm{HSR}}} = 2.85{\kern 2pt} {\mathrm{km}}{\kern 1pt} {\mathrm{s}}^{ - 1}$ and $c_{\mathrm{s}}^{{\mathrm{HSR}}} = 1.37{\kern 2pt} {\mathrm{km}}{\kern 1pt} {\mathrm{s}}^{ - 1}$, respectively (Fig. 3 and Fig. 1c). Therefore, the rupture propagates intersonically with respect to the HSR wave speeds ($c_{\mathrm{s}}^{{\mathrm{HSR}}} < V_{\mathrm{r}} = 2.57{\kern 2pt} {\mathrm{km}}{\kern 1pt} {\mathrm{s}}^{ - 1} < c_{\mathrm{p}}^{{\mathrm{HSR}}}$), in local agreement with basic physics and energy-release-rate analytical models^6,10,11,13,14^ of rupture growth in linear-elastic solids. At several locations along the pressure shock front (Fig. 3, upper inset, cyan star symbols), the local inclination angle *β*_p_ is measured to range between 71° and 83° and, based on the relation (Eq. 1), the corresponding values of the local pressure wave speed, $c_{\mathrm{p}}^{{\mathrm{ISR}}} = V_{\mathrm{r}}{\kern 1pt} {\mathrm{sin}}( {\beta _{\mathrm{p}}} )$, can be obtained and range from 2.43 to 2.54 km s^−1^. When we plot these pressure wave speed values against the strain-rate magnitudes $\left| {{\dot{\boldsymbol \varepsilon }}} \right|^{c_{\mathrm{p}}}$ (from 1.7 × 10^2^ to 4.9 × 10^2^ s^−^^1^) measured at the corresponding locations, we find that they are in excellent correspondence with the viscoelastic response (Fig. 3). These intermediate pressure wave speeds of 2.43 to 2.54 km s^−1^ are also below the rupture speed of *V*_r_ = 2.57 km s^−1^, confirming that the rupture tip travels faster than not only the LSR pressure wave speed but also the pressure wave speeds at the examined locations of the pressure shock front.

It is noteworthy that the effectively supersonic rupture propagation observed in our experiments, in the sense of forming the pressure shock front, cannot be explained by a hyperelastic behavior at the crack tip. Hyperelasticity, in which the stiffening occurs with larger strains (in contrast to the strain-rate effects of viscoelasticity) has been suggested by several numerical studies as a potential mechanism for supersonic crack propagation^12,15,16^. However, the constitutive response of PMMA does not manifest hyperelastic stiffening in tension^32,35^, compression^34–37^ (Supplementary Fig. 3a and b), or shear^40^ experiments for the levels of strains produced by the dynamic cracks in our experiments, which are smaller than 3.2 × 10^−3^ (Supplementary Fig. 3c). In addition, theoretical studies of crack propagation in lattice models^17,41^ suggested that supersonic solutions may exist in the absence of any stiffening, hyperelastic or viscoelastic, near the crack tip. However, our study cannot examine the relevance of those solutions, as the viscoelastic polymers we study do exhibit documented significant viscoelastic stiffening^32,34–37,40^, which is fully consistent with our experimental findings and hence dominates the experimental response.

To summarize, our experimental results capture pressure shock fronts forming in viscoelastic polymers by spontaneously propagating ultra-fast in-plane shear ruptures, and demonstrate the importance of taking into account the non-uniform viscoelastic stiffening in the vicinity of the rupture front to explain the existence and angle of these features. The presented experimentally obtained ruptures are a striking example of how spatially non-uniform local material stiffening and the associated change in energy transfer can completely modify the larger-scale processes, leading to the formation of pressure shock fronts and hence apparent spontaneous supersonic crack propagation. Effectively, the dynamics of the process induces a transient heterogeneity in the elastic properties. The non-uniform strain-rate fields associated with the rupture tip and the resulting non-uniform viscoelastic stiffening are essential for the formation of a pressure shock front, in addition to the shear one. These findings are important for a number of engineering and geological applications, as they demonstrate how high and non-uniform strain rates at the crack tip can induce a non-uniform viscoelastic response in the materials that may be treated as uniformly linear elastic under many other conditions. It is noteworthy that most materials, including rocks^42,43^, exhibit viscoelasticity at the high-strain-rate regimes characteristic of rapidly propagating dynamic cracks. In studies of dynamic earthquake ruptures, the main emphasis so far has been on how high stresses at the rupture tip can induce damage and hence decrease the effective elastic properties and wave speeds^6,44–46^. Our study illustrates the potential of a significant counter-acting phenomenon in which the local elastic properties are transiently increased due to viscoelastic effects, promoting faster rupture propagation, potentially all the way to apparently supersonic ruptures with respect to the wave speeds in most of the bulk.

## Methods

### Laboratory set-up

The laboratory set-up employed in this study is the described in details in previous works^6,7,9,22,24–26^. Our specimen configuration features either a 200 × 250 × 12.5 mm^3^ PMMA or a 200 × 200 × 10 mm^3^ Homalite-100 plate. The sample is separated into two identical halves by an oblique cut at an angle *α* (Fig. 1a and Supplementary Fig. 1a). The juxtaposition of these two halves creates an interface (green-shaded area). In order to obtain repeatable and desired tribological conditions, these surfaces are polished to near optical-grade finish and bead-blasted by employing glass particles in the range of 104–211 mm diameter^7,26^. A uniform load *P* is vertically applied to the specimen, resulting in a resolved normal (*σ*_0 _= *P* cos^2^*α*) and shear (*τ*_0_ = *P* sin *α* cos *α*) stress on the interface. Rupture nucleation is obtained by means of the rapid discharge of an electric potential through a 0.08 mm Ni-Cr wire filament, placed across the interface (Fig. 1a and Supplementary Fig. 1a). Before initiation, electrical charges are accumulated in a capacitor bank in order to achieve a potential of 1.5 (for tests with Homalite-100) to 2 kV (for tests with PMMA). The wire’s rapid sublimation produces a short pressure pulse, inducing the rupture initiation by locally frictionally weakening the interface. In this study, we present three tests conducted on PMMA and one test on Homalite-100. All experiments performed with PMMA have an applied far-field load of *P* *=* 21 MPa; one configuration features an inclination angle of *α* = 30° (Figs. 1 and 2; Fig. 3, top inset; Supplementary Figs. 2 and 3c; and Supplementary Movie 1) and the other two are at *α* = 29° (Fig. 3, bottom inset; and Supplementary Fig. 1). The two tests with *α* = 29° are used to verify the rupture propagation speed (Supplementary Fig. 1), of whom one configuration employs a smaller imaging window in order to achieve higher accuracy with the full-field technique (Fig. 3, bottom inset; and Supplementary Fig. 1a and c); and the other one features an array of three strain gauges (Supplementary Fig. 1). The experiment with Homalite-100 is characterized by a far-field load of *P* = 25 MPa and an inclination angle of *α* = 29° (Fig. 2, right column). The full-field images of velocity, strain, and strain rates are obtained by the employment of our dynamic imaging technique based on the combination of ultra-high-speed photography, DIC algorithms^47^ and post-processing analysis^26^. The strain-rate magnitude field is computed from the strain-rate components as the Frobenius norm of the tensor: $$\left| {{\dot{\boldsymbol \varepsilon }}} \right| = {\dot{\boldsymbol \varepsilon }}_{\mathrm{F}} = \sqrt {{\dot{\boldsymbol \varepsilon }} \\ gt: {\dot{\boldsymbol \varepsilon }}} = \sqrt {\dot \varepsilon _{ij}\dot \varepsilon _{ij}}$$, assuming plane-stress conditions.

### Wave-speed computation

Effective wave speeds due to viscoelastic stiffening are assumed to be a function of the strain rate, by adopting linear-elastic relations with the values of elastic moduli dependent on the local level of strain rate. The elastic modulus of PMMA is tracked as a function of the strain rate using measurements derived from the literature^32,34–37,48–55^ (Fig. 3), ranging from quasi-static compression tests (10^−5^ s^−1^) to highly dynamic conditions (10^4^ s^−1^). As these measurements are from uniaxial tests and they need to be related to the three-dimensional strain-rate fields of our tests, we compute the strain-rate magnitude from the corresponding tensor as $\left| {{\dot{\boldsymbol \varepsilon }}} \right| = \sqrt {\dot \varepsilon _{ij}\dot \varepsilon _{ij}}$. The pressure and shear wave speeds are then calculated as a function of the strain-rate magnitude using the linear-elastic relations for plane strain with the elastic moduli depending on the specific level of strain rate (Fig. 3): $c_{\mathrm{p}} = \sqrt {E\left( {1 - \nu } \right)/\left[ {\rho \left( {1 + \nu } \right)\left( {1 - 2\nu } \right)} \right]}$ and $c_{\mathrm{s}} = \sqrt {E/\left[ {2\rho \left( {1 + \nu } \right)} \right]}$, assuming density *ρ* = 1180 kg m^−3^ (measured) and a constant Poisson’s ratio of *ν* = 0.35^35,37^. Least-square fits of the wave speeds versus strain-rate magnitude (Fig. 3, red line) are then used to determine the LSR and HSR wave-speed values discussed in the main text and presented in Fig. 1c and Supplementary Figure 1c. The functional form of the fitted curve is $a + b{\kern 1pt} {\mathrm{log}}\left( {\left| {{\dot{\boldsymbol \varepsilon }}} \right|} \right)$, where *a* = 2.24 and *b* = 5.9 × 10^−2^ for the pressure wave speed.

### Rupture speed computation

The rupture speed is computed by tracking the rupture tip along the interface using the temporal sequence of the full-field images. In analogy with numerical simulations of shear ruptures^11,56^, we identify the rupture tip as the location where the slip velocity exceeds a preset threshold $\dot \delta _{{\mathrm{th}}}$. The slip velocity $\dot \delta$ is obtained from the difference of the $\dot u_1$ particle velocity component parallel to the interface, immediately above and below it. In our calculations, we use $\dot \delta _{{\mathrm{th}}} = 2.5{\kern 1pt} {\mathrm{m}}{\kern 2pt} {\mathrm{s}}^{ - 1}$ as a threshold for the slip velocity, as it is sufficiently above the noise level to avoid spurious oscillations and still well below the peaks of the slip velocity, which are in the range of 10 to 20 m s^−1^. Changing the threshold within ± 1 m s^−1^ does not produce a substantial difference in the arrival times. Adopting this procedure, the rupture arrival time is identified at each location along the interface and the rupture speed is computed with a second-order-accurate central-difference scheme, using the sequence of locations and rupture arrival times (Fig. 1c and Supplementary Fig. 1c). To validate the rupture speed obtained from this procedure, we also compute it from the arrival times at a set of three strain-gage measurement locations, each measuring the direct strain in the direction parallel to the interface, *ε*_11_ (Supplementary Fig. 1). In this calculation, we select the threshold of the strain signal to be $\left| {\varepsilon _{11,{\mathrm{th}}}} \right| = 10^{ - 3}$ (Supplementary Fig. 1b, horizontal dashed line). Two nominally identical experiments have been conducted on PMMA, under a far-field load of *P* = 21 MPa and an inclination angle of *α* = 29°. The rupture speed has been measured using either the DIC technique (employing a small field of view) or the strain gages (Supplementary Fig. 1). (Strain measurements by DIC and strain gages cannot be performed simultaneously in our experiments, as the high-power flash illumination, required for the high-speed image acquisition^26,57^, releases a strong electro-magnetic pulse that interferes with the strain gages, compromising their ability to measure physical strains.) The electric discharge, delivered to the Ni-Cr wire for triggering, also induces an electro-magnetic pulse that last several tens of microseconds. This disturbance produces spurious oscillations that overlap with the strain signals, in particular in the proximity of the wire notch (Supplementary Fig. 1a and b). However, the main features associated with rupture propagation are still clearly identifiable and allow precise rupture arrival time calculations, the results of which are in excellent agreement with the DIC ones (Supplementary Fig. 1c).

## Electronic supplementary material

Supplementary Movie 1

Supplementary Information

Description of Additional Supplementary Files

## Data Availability

Data supporting the findings of this study are available from the corresponding author upon request.
