# Supplementary material for: Pressure shock fronts formed by ultra-fast shear cracks in viscoelastic materials
Source: Nat Commun. 2018 Nov 12;9:4754. doi: 10.1038/s41467-018-07139-4 (PMC6232150; doi:10.1038/s41467-018-07139-4)
Supplement: Supplementary file 2 — Supplementary Information [file 41467_2018_7139_MOESM2_ESM.pdf]

Supplementary Information:

Pressure shock fronts formed by ultra-fast shear cracks in viscoelastic materials

Gori et al.

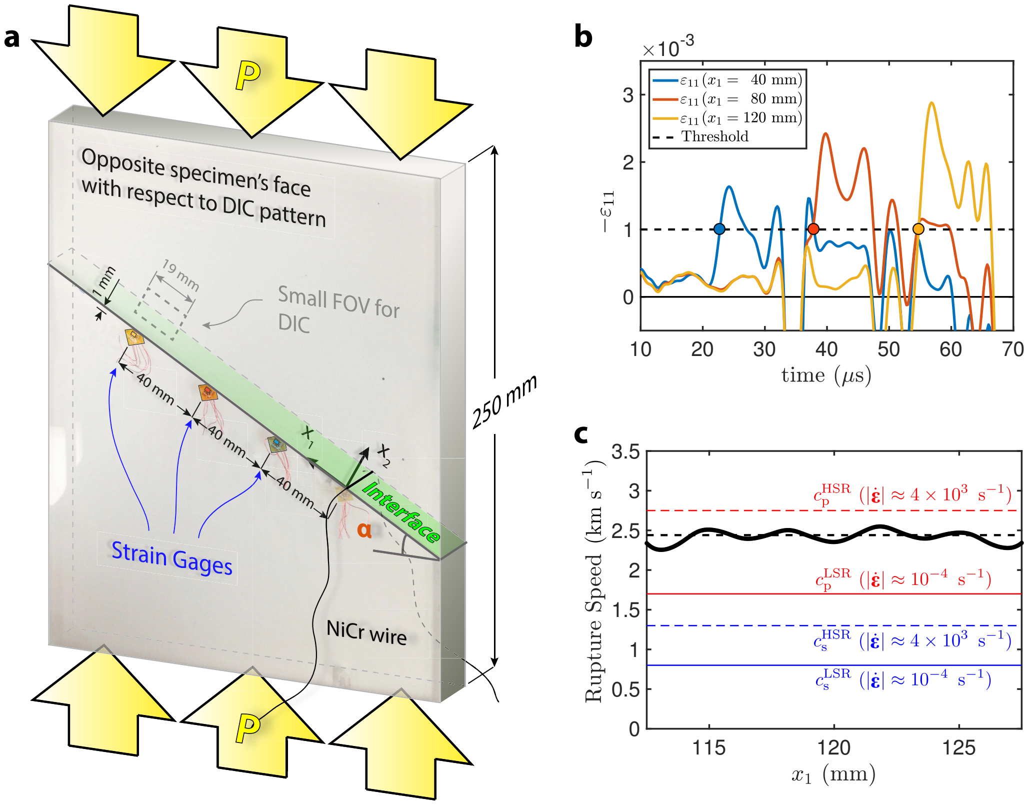

Supplementary Figure 1. Rupture speed computation via DIC and strain gage measurement techniques. Two nominally identical tests are conducted using either the DIC technique or the strain gages. **a**, Back-side view of the PMMA sample, where an array of three strain gages has been applied along the interface. These strain stations are positioned approximately 40 mm away from each other, with the first one 40 mm away from the wire's location. The field-of-view window of the speckled pattern is applied on the front side of the specimen and is indicated by a dashed rectangle ($19 \times 12\ \mathrm{mm}^2$). **b**, Time history of the direct strain in the direction parallel to the interface $-\varepsilon_{11}$, measured at the three locations shown in Supplementary Fig. 1a. The color of each strain signal matches that of the corresponding locations in Supplementary Fig. 1a. The transit of the rupture is associated with the initial peaks, where positive sign of $-\varepsilon_{11}$ indicates compression, in accordance with the right-lateral motion of the rupture. The rupture arrival time has been computed considering a threshold of $|\varepsilon_{11,\mathrm{th}}| = 10^{-3}$ (horizontal dashed line). **c**, Rupture speed computed using the full-field velocity maps obtained with DIC over the small field of view indicated in **a**. The pressure (red lines) and shear (blue lines) wave speeds are reported, where the LSR and HSR conditions correspond to the solid and dashed lines, respectively. The black horizontal dashed line represents the rupture speed averaged between the three strain-measurement stations.

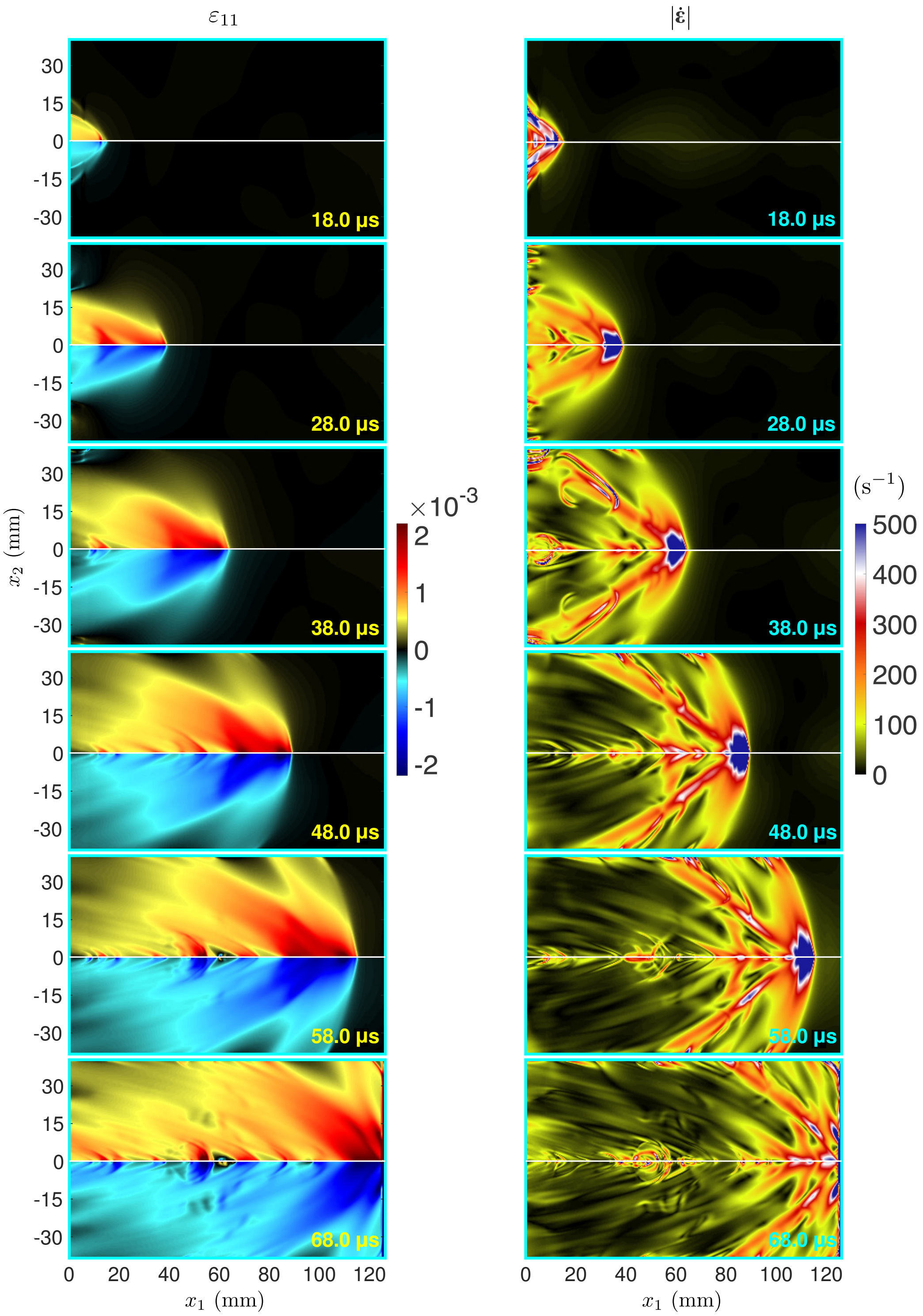

Supplementary Figure 2. Snapshots of full-field images showing the progression of the rupture in PMMA. Left column: strain component in the direction parallel to the interface, ε_{11}. **Right column:** strain rate magnitude $|\dot{\varepsilon}|$. The collection of images is from the large field of view ($128 \times 80\ \mathrm{mm}^2$) and, as the rupture propagates from left to right in the positive x_1-direction, each image corresponds to a snapshot from 18 to $68\ \mu\mathrm{s}$, every $10\ \mu\mathrm{s}$. In analogy with Fig. 2, a double pair of shock fronts is clearly discernible, as they become well developed in the later frames.

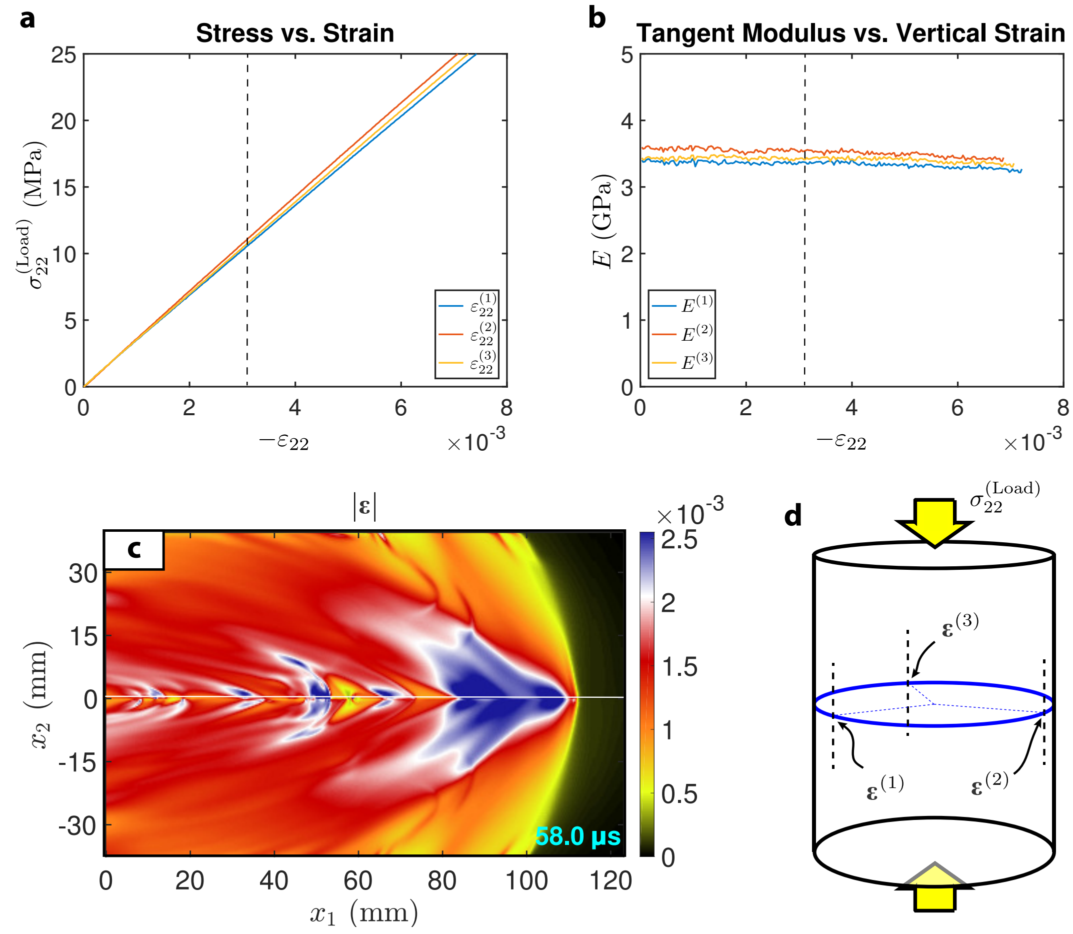

Supplementary Figure 3. No hyperelasticity in PMMA for strains of interest. a, In a quasi-static unidirectional compression test in PMMA, $|\dot{\varepsilon}| \approx 10^{-4}\ \mathrm{s}^{-1}$, the vertical compressive stress shows a linear dependence on the vertical strain, up to strains of 7×10^{-3}. **b**, The Young's modulus, computed as the local tangent of the stress-strain curve, does not exhibit stiffening with strain. **c**, The full-field equivalent strain $|\varepsilon|$ shows that our propagating dynamic cracks produce levels of strain smaller than 3.2×10^{-3}, which would fail to activate hyperelastic effects. **d**, Setup employed to produce the results presented in a and b. Three strain gages equally spaced around the circumference (blue line) of a PMMA cylinder (from the same material as that for the dynamic rupture experiments) measure the vertical component of strain as the load is applied vertically (yellow arrows).
