# Supplementary material for: Pressure shock fronts formed by ultra-fast shear cracks in viscoelastic materials
Source: Nat Commun. 2018 Nov 12;9:4754. doi: 10.1038/s41467-018-07139-4 (PMC6232150; doi:10.1038/s41467-018-07139-4)
Supplement: Supplementary file 3 — Description of Additional Supplementary Files [file 41467_2018_7139_MOESM3_ESM.pdf]

Description of Additional Supplementary Files

File Name: Supplementary Movie 1

Description: Rupture with pressure and shear shock fronts. The temporal evolution of the full-field volumetric strain rate, $|\dot{\boldsymbol{\varepsilon}}|$, is shown for the experiment with PMMA discussed in the text ($P = 21$ MPa, $\alpha = 29°$), with a field of view of $128 \times 80\ \mathrm{mm}^2$. The time is in microseconds and starts at the rupture nucleation. The movie displays the rupture progression as it develops from left to right in the positive x_1-direction. The formation of both the pressure and the shear shock fronts is visible in the early frames ($t < 42\ \mu\mathrm{s}$), and becomes well developed as the rupture advances ($t \geq 42\ \mu\mathrm{s}$). The upper half-portion of the pressure shock front is extensional, while the lower portion is compressional, in accordance with the rightwards propagation of the left-lateral in-plane shear rupture. A snapshot taken from this sequence, at $t = 58\ \mu\mathrm{s}$, is reported in Fig. 2c. The presence of both the pressure and shear shock fronts indicates the supersonic nature of the rupture.
